# Biopolymeric Blends of Thermoplastic Starch and Polylactide as Sustainable Packaging Materials

**DOI:** 10.3390/polym16091268

**Published:** 2024-05-01

**Authors:** Antun Jozinović, Mario Kovač, Vesna Ocelić Bulatović, Dajana Kučić Grgić, Martina Miloloža, Drago Šubarić, Đurđica Ačkar

**Affiliations:** 1 Faculty of Food Technology Osijek, Josip Juraj Strossmayer University of Osijek, Franje Kuhača 18, 31000 Osijek, Croatia; ajozinovic@ptfos.hr (A.J.); dsubaric@ptfos.hr (D.Š.); 2 Faculty of Agriculture and Food Technology, University of Mostar, Biskupa Čule bb, 88000 Mostar, Bosnia and Herzegovina; mario.kovac11@gmail.com; 3 Faculty of Chemical Engineering and Technology, University of Zagreb, Trg Marka Marulića 19, 10000 Zagreb, Croatia; dkucic@fkit.unizg.hr (D.K.G.); miloloza@fkit.unizg.hr (M.M.)

**Keywords:** biomaterials, potato starch, polylactic acid, citric acid, blends, biodegradation

## Abstract

The improper disposal of plastics is a growing concern due to increasing global environmental problems such as the rise of CO_2_ emissions, diminishing petroleum sources, and pollution, which necessitates the research and development of biodegradable materials as an alternative to conventional packaging materials. The purpose of this research was to analyse the properties of biodegradable polymer blends of thermoplastic potato starch (TPS) and polylactide, (PLA) without and with the addition of citric acid (CA) as a potential compatibilizer and plasticizer. The prepared blends were subjected to a comprehensive physicochemical characterization, which included: FTIR-ATR spectroscopy, morphological analysis by scanning electron microscopy (SEM), determination of thermal and mechanical properties by differential scanning calorimetry (DSC), water vapour permeability (*WVP*), as well as biodegradation testing in soil. The obtained results indicate an improvement in adhesion between the TPS and PLA phases due to the addition of citric acid, better homogeneity of the structure, and greater compatibility of the polymer blends, leading to better thermal, mechanical and barrier properties of the studied biodegradable TPS/PLA polymer blends. After conducting the comprehensive research outlined in this paper, it has been determined that the addition of 5 wt.% of citric acid serves as an effective compatibilizer and plasticizer. This supplementation achieves an optimal equilibrium across thermal, mechanical, morphological, and barrier properties, while also promoting material sustainability through biodegradation. In conclusion, it can be stated that the use of thermoplastic starch in TPS/PLA blends accelerates the biodegradation of PLA as a slowly biodegradable polymer. While the addition of citric acid offers significant advantages for TPS/PLA blends, further research is needed to optimize the formulation and processing parameters to achieve the desired balance between mechanical strength, thermal and barrier properties and biodegradability.

## 1. Introduction

The staggering global consumption of plastics has reached an alarming 8.3 billion tons over the last seven decades, of which the packaging sector accounts for a significant 40% [1,2,3]. The high consumption suggests a frightening scenario of an estimated 12 billion tons of long-lasting plastic waste by 2050, contributing significantly to environmental pollution and exacerbating the problem of microplastics [2,4]. An enormous amount of 150 million tons of plastics is annually used for single-use packaging, further increasing the environmental impact. Worryingly, a colossal 8 million tons of plastics enter the oceans every year, exacerbating marine pollution problems. Conventional plastics, which are primarily derived from petroleum, pose an enormous challenge due to the deterioration of mechanical properties during reprocessing and recycling, necessitating a constant dependence on petrochemical raw materials [5]. To tackle this looming crisis, an innovative approach is essential. The use of biopolymers from agriculture, particularly starch and cellulose, offers a promising solution. Not only do these biopolymers come from renewable resources, but they also have the advantage of being easily biodegradable. Rapid technological advances, particularly in the study of microstructures at different levels and the understanding of the intricate relationships between structures and properties, have driven the development of environmentally friendly materials. This newfound understanding not only helps to solve environmental problems but also opens up unprecedented opportunities to produce materials tailored for novel applications, ushering in a promising era of sustainable innovation.

Starch, a natural carbohydrate storage material found in plant granules, is a biodegradable and annually renewable resource. It is not only an environmentally friendly, but is also economically attractive due to its availability and low cost [6,7,8]. Extensive research and practical applications are currently focusing on the thermoplastic modification of starch. In this process, native starch is mixed with plasticizers at elevated temperatures and under specific shear stress. The aim is to change the original starch structure, either partially or completely, primarily by breaking the crystallinity and the intra- and intermolecular hydrogen bonds [8]. In practical applications, thermoplastic starch (TPS) is often used in the form of blends with other polymers. The reason for this approach lies in the inherent properties of pure TPS; it tends to be soft, overly sensitive to moisture and exhibits exceptionally rapid biodegradability. To overcome these challenges, most researchers choose to blend TPS with other biodegradable polymers. These include polylactic acid (PLA) [9,10], polybutylene succinate (PBS) [11,12,13], polybutylene adipate-co-terephthalate (PBAT) [14,15], polyvinyl alcohol (PVA) [16], polycaprolactone (PCL) [17,18], and polyglycolic acid (PGA) [19]. This strategic combination not only improves the mechanical properties of TPS, but also mitigates its sensitivity to moisture, resulting in a more versatile and robust material suitable for various applications.

PLA (polylactic acid), a representative synthetic polymer derived from biologically produced monomers, is compatible with numerous conventional thermoplastic processing techniques. Despite this versatility, its widespread use is hindered by its excessive cost. The pursuit of biodegradable polymeric materials has become a focus of current research and has resulted in several products being available on the market. However, the transition to replacing conventional polymers, especially in promising areas such as packaging and mulch films, is hampered by either suboptimal performance or prohibitive costs [20]. While PLA tends to be pricier than conventional petroleum-based polymers, especially for disposable or short-term applications, blending PLA with TPS offers a promising solution [8]. This blend not only reduces the cost of PLA but also expands the potential applications of TPS. Moreover, by adjusting the starch content in the blend, the degradation rate of PLA can be effectively controlled. However, hydrophilic starch, with a great number of hydroxyl groups, and hydrophobic PLA, with hydroxyl and carboxyl end groups, often result in poor interfacial adhesion between them. In order to overcome these drawbacks, certain non-toxic functional additives are required to improve the properties of PLA/starch films [21].

The primary objective of the present study was to acquire comprehensive insights into the characteristics of TPS/PLA blends, as well as to evaluate the impact of citric acid (CA) as a compatibilizer on their morphological composition, mechanical attributes, and thermal properties. Citric acid enhances TPS plasticization and melt processing, potentially accelerating the fragmentation and dissolution of starch granules as well as the fluidity of TPS. Co-plasticizing starch with a blend of glycerol/citric acid proves intriguing due to its capacity to enhance starch plasticization, potentially enabling partial esterification and yielding lower molecular weight chains [22]. However, it is important to point out that citric acid (2-hydroxypropane-1,2,3 tricarboxylic acid, C_6_H_8_O_7_) itself is recognized as a safe food additive (GRAS) and is used widely in food, cosmetics, and pharmaceutical industries, deemed safe for both human health and the environment [23,24,25].

## 2. Materials and Methods

### 2.1. Materials

Polylactic acid (PLA), trademark IngeoTM Biopolymer 4043D, (d-isomer content = 4.3% as declared by the producer with melt flow rate (MFR) of 6.0 g/10 min (at 210 °C/2.16 kg), density of 1.24 g/cm^3^, molecular weight of Mw = 168,000 g/mol, melting temperature in the range of 145–160 °C, and the glass transition point between 55 and 60 °C, was procured from Nature Works LLC, Minnetonka, MN, USA. The native potato starch used in this study was starch isolated from a potato cultivar with code name SL 13–25. The isolation procedure and characterization of starch are described in our previous paper [26].

Reagent grade glycerol (99.8% purity), used to plasticize native potato starch, was obtained from Gram-Mol, Zagreb, Croatia. Citric acid monohydrate (p.a.) (CA) was supplied by Gram-Mol, Zagreb, Croatia.

#### 2.1.1. Preparation of Thermoplastic Starch and Thermoplastic Starch–Citrate

Thermoplastic starch (TPS_SL) was prepared by blending native potato starch (potato cultivar SL 13–25) with glycerol added as a plasticizer with a ratio of 60 (starch)/40 (glycerol). The TPS_SL was extruded in a laboratory single-screw extruder (Model 19/20DN; Brabender GmbH, Duisburg, Germany) with a 4 mm round die. The barrel temperatures of the extruder were set at 100 °C, 100 °C and 130 °C for the first (dosing), second (compression) and third (discharge) zones, respectively. The extruded samples were cut and granulated into a pellet form. Potato starch citrates were prepared by the methodology of Kapelko-Zeberska et al. [27] with the application of 5 wt.%, 10 wt.% and 20 wt.% of citric acid. TPS_SL-citrates (label TPS_SL_XCA, where X is the content of citric acid) were obtained by the same procedure as TPS_SL, using glycerol as a plasticizer, in a proportion of 40 wt.%.

#### 2.1.2. Preparation of Binary Blends

After the preparation of TPS_SL and TPS_SL-citrate, the blends with PLA were also prepared using a two-step process. Thermoplastic starch was dried at 105 °C for 24 h to remove residual moisture. In the first step, the main components of the blends were mixed manually in polyethylene bags for 5 min (varying amounts of TPS_SL and TPS_SL-citrates (40, 50 and 60 wt.%) with PLA pellets). The components were added manually to the Brabender kneader, preheated to 170 °C, with a rotor speed of 60 rpm for 5 min. Finally, the prepared homogeneous blends were moulded in a laboratory hydraulic press (Fontune, Holland (SRB 140, EC 320 × 320 NB)) at a temperature of 180 °C, a pressure of 25 kPa for 5 min, with a preheating time of 1 min and cooling time of 20 min. The blends were then cooled to ambient temperature while remaining under pressure before being removed from the hydraulic press. Pure PLA, TPS_SL, and TPS_SL-citrate were treated under the same mixing conditions. The dimensions of the mould varied based on the intended investigation. For assessing mechanical, thermal and structural properties, the dimensions were set at 10 mm × 10 mm × 1 mm. However, for studying biodegradation in soil, monitoring the process, and conducting subsequent morphological and spectroscopic analyses, the granules of the samples were compressed between two plates to create thin film resembling sheets, with a thickness of about 0.4 mm. The thickness of the samples depended on the proportion of TPS; higher TPS proportions resulted in thicker films. These films were then cut into square moulds with dimensions of 15 mm × 15 mm and weighed on an analytical balance (AEAAK45, SAB 125i, Adam Equipment Co. Ltd., Milton Keynes, UK) with a precision of 0.00001 g.

### 2.2. Chemical Structure Characterization

Attenuated total reflectance Fourier transformed infrared spectroscopy (ATR-FTIR) of the films was measured on a PerkinElmer (Waltham, MA, USA) Spectrum one FT-IR spectrometer using a diamond crystal. Four recordings were performed for each sample and an average spectrum was evaluated from these recordings. The spectra were obtained at a resolution of 4 cm^−1^ in the range of 4000 cm^−1^ to 650 cm^−1^.

### 2.3. Morphology

The morphology of the prepared samples and fractured surfaces was examined by scanning electron microscopy (SEM). SEM was carried out using a Vega 3 scanning electron microscope (Tescan, Brno, Czech Republic) with an operating voltage of 20 kV. The samples were mounted on small stubs and coated with a thin layer of gold using a sputter coater to improve the conductivity.

### 2.4. Differential Scanning Calorimetry Analysis (DSC)

Thermal properties evaluation was performed by differential scanning calorimetry, model Mettler Toledo DSC 822e (Greifensee, Switzerland). The samples were loaded onto an aluminium pan covered by an aluminium lid. The weight of samples varied between 9.0 mg and 11.0 mg. Two heating cycles were used: first the samples were heated in the temperature range from 25 °C to 250 °C under nitrogen atmosphere (50 mL min^–1^) at a heating rate of 10 °C min^–1^; they were kept at 250 °C for 5 min to eliminate the thermal history, and then cooled to −100 °C at a cooling rate of 10 °C min^–1^, and immediately reheated to 250 °C. Nitrogen (99.99% purity) was used to purge the thermal analysis system at a rate of 50 mL min^−1^. The thermograms refer to the second heating. The crystallinity of PLA in the blend was calculated, where the fusion heat of 100% crystallinity of PLA (Δ*H_m_*(PLA)) was set equal to 93.1 J/g [28].

### 2.5. Water Vapour Permeability (WVP)

Water vapour permeability (*WVP*) of the blends was performed using Herfeld’s apparatus [28,29]. Herfeld’s apparatus consists of a glass container, filled with 50 mL of distilled water, closed with a metal ring containing a circular opening with a diameter of 36 mm. The test samples were placed face down on the lid of the 55 mm diameter jar and sealed with the metal ring. The glass jar was placed in a desiccator containing 97% sulphuric acid (p.a.). The weight of the glass container with the test samples and water was determined at the beginning and after the specified time interval of 24 h (one day) and 48 h (two days). The water vapour permeability (*WVP*) was determined according to Equation (1):

*WVP*/g m^−2^day^−1^ = ((*m*_0_ − (*m*_2_ + *m*_3_)/2)/*A* (1)

where *m*_0_ is the mass of the device with water and specimen at the beginning (g), *m*_2_ is the mass of the device with water and a test tube after 24 h (g day^−1^), *m*_3_ is the mass of the device with water and a test tube after 48 h (g day^− 1^), *A* = r^2^π (m^2^) is the film surface of the examined sample, and r (m) is the radius of the film, permeation area, respectively.

### 2.6. Tensile Test of the Materials

The tensile properties of the films were determined with a Zwick 147670 Z100/SN5A Universal Test instrument (Ulm, Germany), equipped with a 2000 N load cell at 23 °C and 65% relative humidity, following the ISO 527-1:2019 standard [30]. For the tensile tests, a crosshead speed of 50.00 mm min^–1^ was used. The sample’s thickness was collected by taking the average values of three measurement points across each sample. For statistical purposes, a minimum of five specimens per sample were tested, and average values were calculated, with a standard deviation of less than 5%.

### 2.7. Determination of Biodegradability of Materials in Soil

**Figure 1:**
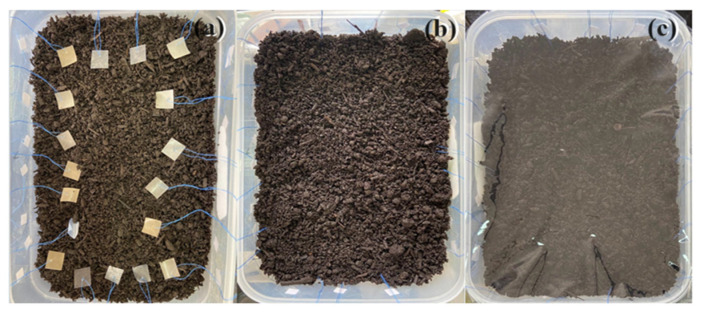
Preparation of samples for the biodegradation test in soil: (**a**) samples arranged on 300 g of soil in a container; (**b**) samples covered with another 300 g of soil; (**c**) container covered with foil.

The biodegradability test of the prepared materials was carried out following ISO 17556:2019 standard [31]. The materials were placed in reactors containing 300 g of moist soil (60% RH). The test samples were carefully placed in plastic containers, after which they were covered with an additional 300 g of moist soil and protected with foil to maintain soil moisture. The process of preparing biodegradation of blends is shown in Figure 1. To facilitate identification, the samples were labelled before they were removed from their containers. Prior to testing, the soil was enriched with a mixed microbial community of bacteria and fungi, including *Pseudomonas aeruginosa*, *Bacillus subtilis*, *Bacillus cereus*, *Candida* sp., *Trichoderma reesei* and *Aspergillus niger*. These microorganisms were isolated from environmental samples and are kept in the collection of the Department of Industrial Ecology of the University of Zagreb Faculty of Chemical Engineering and Technology. The soil was moistened with deionized water every day. Samples of the blends were placed in a thermostat set at 58 °C. Samples were taken at seven-day intervals, on the 7th, 14th, 21st, 28th, 42nd and 56th day. After extraction from the soil, the samples were washed with 70% ethanol (p.a) and deionized water to remove organic matter and soil aggregates, and were then air-dried for four days before weighing.

An insight into the biodegradation of the prepared samples was obtained by monitoring the mass change at intervals of seven days. The mass change percentage of the materials after biodegradation was calculated using the following expression, Equation (2):

mass loss (%) = (*m_before_* − *m_after_*)/*m_before_* × 100 (2)

where *m_before_* (g) is the weight of the materials before biodegradation and *m_after_* (g) is the weight of the materials after biodegradation. The obtained values are the average result of three samples treated under the same conditions. The biodegradation phenomenon was evaluated through mass loss and analysed by monitoring morphological change (polarized optical microscope) and structural analysis (FTIR-ATR).

### 2.8. Polarizing Optical Microscope (POM)

A polarizing optical microscope (Olympus BX53M polarizing optical microscope, Tokyo, Japan) was employed to determine the morphologies of TPS/PLA and TPS-citrate/PLA blends before and after biodegradation in soil. Figures were collected at 50 objective magnifications.

## 3. Results and Discussion

### 3.1. Characterization of Materials

#### 3.1.1. Results of FTIR—ATR Spectroscopy

**Figure 2:**
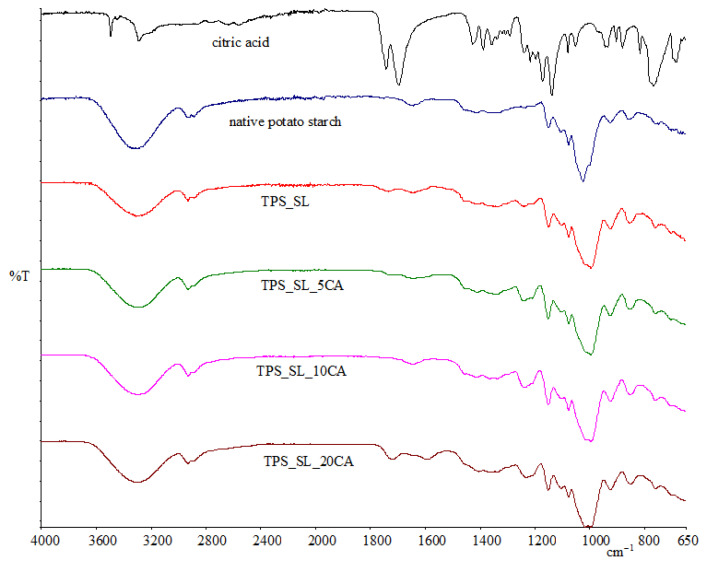
FTIR spectra of citric acid, native potato starch, TPS_SL and TPS-SL-citrate.

FTIR analysis was used to classify the chemical bonds present within the structure of the prepared blends. The signals obtained from the infrared spectrum were compared with characteristic binding segments previously described in the literature for native potato starch, thermoplastic starch and ester groups. However, if two polymers are compatible, a notable chemical interaction arises, characterized by the presence of distinct features in the FTIR spectra of the blends. These features may include shifts in bands and broadening, indicative of interactions such as hydrogen bonding or dipolar interactions between the polymer chains. So, FTIR can be used to identify segment interactions and provide information about the phase behaviour of polymer blends. The aim of the analysis was to determine whether the addition of citric acid to native potato starch leads to esterification. Figure 2 shows the FTIR spectrum of native potato starch, TPS_SL and TPS_SL-citrate. The first maximum, which occurs around 3300 cm^−1^ is characteristic of bound water or moisture in the sample and is visible in all analysed samples; it is related to the pronounced hydrophilic character of TPS_SL or native potato starch. The appearance of this band indicates the vibrational stretching of the inter- and intra-molecular bonds between the hydroxyl groups of the starch and the plasticizer glycerol. In this example, it is a single O-H bond, a hydroxyl group, which can be recognized by the stretching, which, according to the literature, corresponds to the stretching of the hydroxyl group from 3300 cm^−1^ to 3400 cm^−1^.

The spectral signature of the OH group is characterized by a broad band [32]. Referring to Figure 2, for all samples the band at 2800–3500 cm^−1^ is evident, which can be attributed to the C-H stretching of the starch moieties, as well as of citric acid (for comparison, the FTIR spectrum of citric acid is also depicted in Figure 2). The bands of free OH groups in the citric acid spectrum yield a band with a maximum at 3496 cm^−1^, while bands of the OH groups involved in intramolecular and intermolecular hydrogen bonds are observed at 3448 cm^−1^ and 3288 cm^−1^, respectively. Native potato starch exhibits a band, around 2800–3000 cm^−1^, corresponding to the stretching of the C-H bond, as well as a band of the C-O bond at 1025 cm^−1^ which is related to the glycosidic linkages between glucose units in starch molecules, indicative of the polysaccharide backbone (Figure 2). Upon comparing the FTIR spectra of native potato starch and TPS_SL (Figure 2), several notable differences are observed. In the spectrum of TPS_SL, stronger and more pronounced bands are visible at 2854 cm^−1^. Furthermore, the double peak in the region of 1600–1750 cm^−1^, characteristic of native potato starch, has transformed into a single peak at 1648 cm^−1^ with pronounced intensity. These changes can be attributed to the addition of glycerol plasticizer and the presence of starch-bound water molecules, respectively [33,34]. When the FTIR spectra of native potato starch and TPS_SL are compared, an increase in the intensity of the maximum at 2930 cm^−1^ can be observed, which indicates the stretching of the C-H bond in the CH_2_ group. The maximum at 1025 cm^−1^ indicates the presence of anhydrous glucose units (C-O-H) in the starch molecule. The FTIR spectrum of TPS_SL_5CA shows the appearance of new maxima attributed to the addition of a carboxylic acid, and maxima indicating the stretching of the C=O bond of the carbonyl group appear at 1731 cm^−1^, while peaks are observed at 1241 cm^−1^ and 1211 cm^−1^ evidencing the stretching of the C-O bond of the ester, indicating that esterification had occurred with the addition of citric acid to the starch. Also, the peak intensity of hydroxyl groups of TPS at 3300 cm^−1^ significantly decreased as the CA content increased, suggesting that the esterification process between hydroxyl groups of TPS and carboxylic acid groups of CA had occurred to form ester bonds in modified TPS. The appearance of new maxima, which are characteristic of the bonds of the ester group, indicates that the addition of citric acid to TPS_SL has led to esterification.

**Figure 3:**
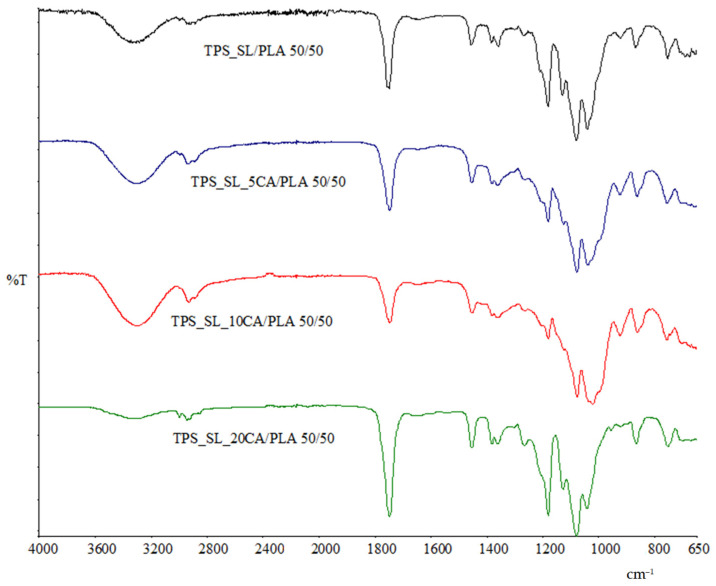
FTIR spectra of TPS_SL/PLA 50/50 and TPS_SL-citrate/PLA 50/50 polymer blends.

The FTIR spectra of all investigated TPS_SL/PLA polymer blends exhibit characteristic peaks that are consistent with those observed in both TPS_SL and PLA spectra (Figure 2 and Figure 16). From Figure 3, it is discernible that a broad band spanning 3600–3000 cm^−1^ is present, indicative of the stretching vibrations of the hydroxyl group (-OH), which exhibit heightened intensity with increasing proportions of citric acid. Nonetheless, it is noteworthy that at a concentration of 20 wt% citric acid, there is a significant decrease in intensity. Furthermore, all examined blends showcase a conspicuous peak at 1748 cm^−1^, corresponding to the stretching vibrations of the carbonyl group (C=O) within the polyester. Additionally, characteristic stretching signals originating from the -CH group are observable at approximately 3000 cm^−1^, emanating from both PLA, TPS, and TPS-citrate. Upon testing polymer blends with citric acid, the anticipated TPS_SL-citrate-specific peaks were also detected (Figure 3). Notably, the spectra display varying intensities of these individual peaks, reflecting both the increasing concentration of citric acid and the differing proportions of the constituent components within the polymer blend.

#### 3.1.2. Scanning Electron Microscopy (SEM) Results

**Figure 4:**
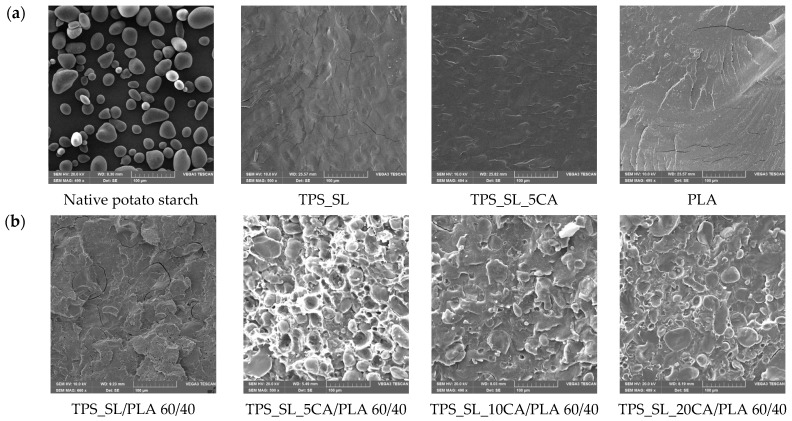

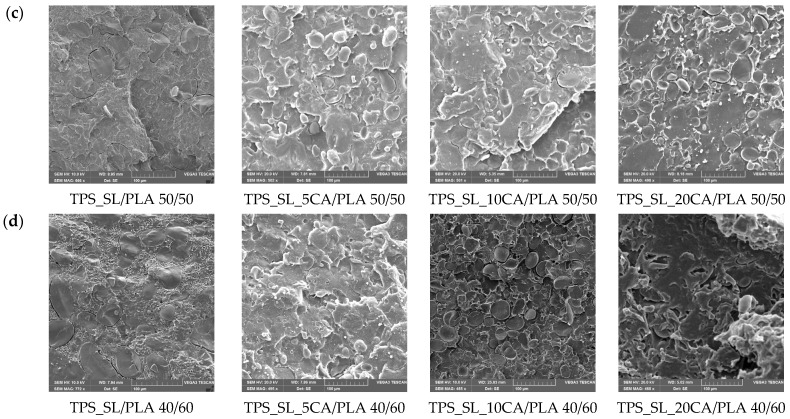
SEM micrographs of: (**a**) Native potato starch, TPS_SL, TPS_SL_5CA and PLA; (**b**) TPS_SL/PLA and TPS_SL-citrate/PLA 60/40; (**c**) TPS_SL/PLA and TPS_SL-citrate/PLA 50/50; (**d**) TPS_SL/PLA and TPS_SL-citrate/PLA 40/60.

The morphological structure of the fracture surfaces of the TPS_SL/PLA blends were examined with a scanning electron microscope, and the resulting SEM micrographs are shown in Figure 4.

Figure 4a shows an SEM micrograph of native potato starch, which shows a large number of spherical granules of different sizes that tend to agglomerate. The SEM micrograph of TPS_SL shown in Figure 4a indicates that the spherical starch granules dissolve due to shearing and temperature action on the starch during the plasticization process; the visible starch ring decreases, more precisely, the starch granules dissolve, and a homogeneous and compatible structure is formed. It can be concluded that the plasticization with glycerol was successful and that the furrows visible on the micrograph of TPS_SL were caused by the brittle fracture of the material during the preparation of the sample for SEM analysis. In Figure 4a, the homogeneous and continuous morphology of pure polylactide can also be seen. The flat and smooth surface of PLA correlates with its known mechanical properties, namely high breaking strength, low elasticity, and firmness as well as a low value of elongation at break.

The morphological structure of the thermoplastic starch obtained from starch citrate, i.e., TPS_SL_5CA, indicates good compatibility, which is due to the formation of an ester bond, i.e., the chemical reaction of citric acid and –OH groups from starch (Figure 4a).

SEM micrographs of polymer blends TPS_SL/PLA 60/40 (Figure 4b), TPS_SL/PLA 50/50 (Figure 4c) and TPS_SL/PLA 40/60 (Figure 4d) show two different phases in which TPS_SL remained dominant as a dispersed phase in the PLA matrix. The TPS_SL granules were separated from the PLA matrix indicating that TPS, which was not obtained from starch citrate, does not improve the inter-phasal interaction between hydrophobic PLA and hydrophilic TPS_SL. The large volume of pronounced voids and phase separation indicates incompatibility between TPS_SL and PLA, and the visible boundary between TPS_SL and the PLA matrix indicates weaker penetration of the dispersed TPS_SL particles into the PLA matrix due to poor adhesion to the interfacial surface. This consequently results in poorer mechanical properties of the polymer blend as the stress transfer between the polymer matrix and the dispersed particles is weakened. It is observed that with the increase in the proportion of TPS_SL in polymer mixtures, the size of the TPS_SL phase also increases, which leads to the accretion or coalescence of TPS particles.

All the micrographs of the polymer blends obtained with TPS_SL-citrate show that with the increase in the proportion of citric acid, TPS_SL spherical granules are better dissolved, which is why they are less visible and penetrate the polylactide matrix more easily. The citric acid leads to improved adhesion between TPS_SL-citrate and the PLA matrix, and the best compatibility was observed with the polymer mixture TPS_SL_10CA/PLA 60/40 (Figure 4b). The fine particles of TPS_SL are well embedded in the polylactide matrix, whereby the size of the visible particles of TPS_SL has decreased. In the case of the polymer blend TPS_SL_10CA/PLA 60/40 (Figure 4b), a decrease in voids and an improvement in adhesion can be observed compared to TPS_SL_20CA/PLA 60/40 (Figure 4b), which leads to the conclusion that with an increase in the proportion of citric acid, adhesion is weakened.

#### 3.1.3. Differential Scanning Calorimetry (DSC) Results

**Figure 5:**
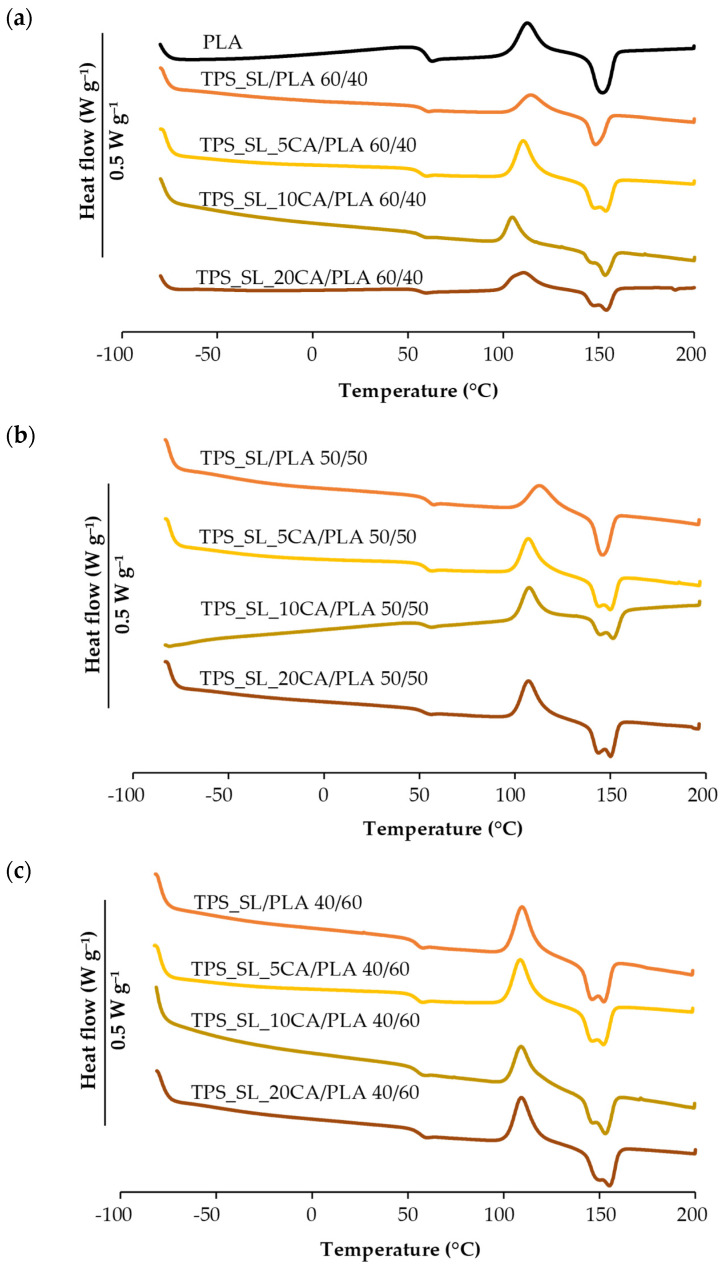
DSC thermograms of biopolymeric blends: (**a**) TPS_SL/PLA and TPS_SL-citrate/PLA 60/40; (**b**) TPS_SL/PLA and TPS_SL-citrate/PLA 50/50; (**c**) TPS_SL/PLA and TPS_SL-citrate/PLA 40/60.

Differential scanning calorimetry was performed with the aim of determining the influence of TPS and citric acid as a compatibilizer on the phase transitions of PLA. From the DSC thermograms of polymer blends TPS_SL/PLA, TPS_SL_5CA/PLA, TPS_10_CA/PLA and TPS_SL_20CA/PLA in the ratios, 60/40, 50/50 and 40/60, characteristic phase transitions were determined: melting temperatures, *T_m_* (°C), melting enthalpy, Δ*H_m_* (Jg^−1^), crystallization temperature, *T_c_* (°C), enthalpy of crystallization, Δ*H_c_* (Jg^−1^), and the degree of crystallinity, *χ_c_* (%). Table 1 shows the obtained values of characteristic temperatures and enthalpies and the calculated degree of crystallinity for PLA, TPS_SL/PLA, and TPS_XCA/PLA. DSC thermograms of the second heating cycle of pure PLA and TPS_SL/PLA polymer blends are shown in Figure 5.

According to the values from the literature, the glass transition temperature (*T_g_*) of pure PLA is 58.5 °C (Table 1), which is also visible on the DSC curves (Figure 5a) [35]. The glass transition temperature shows that the polymer chains of polylactide are in a glassy, or more precisely low-energy state, at room temperature, where the groups oscillate around their equilibrium position. The addition of thermoplastic starch lowers the glass transition temperature by approximately 2–3 °C compared to the *T_g_* (°C) of pure polylactide. Greater mobility of polymer chains occurs due to the lowering of the glass transition temperature, which leads to improved miscibility of PLA and TPS_SL blends [8]. The glass transition temperature does not change significantly with an increase in the proportion of TPS in polymer mixtures, while the addition of citric acid further lowers *T_g_* (°C), which indicates an improvement in the miscibility of the investigated polymer blends using TPS_SL-citrates. With the subsequent heating of polylactide, the activity of macromolecules within the chain increases, which leads to the process of restructuring and energy release [36]. As a result of cold crystallization, an exothermic change was determined on the DSC curve, visible by the appearance of the maximum peak on the curve at 112.4 °C (Figure 5a), which indicates the cold crystallization temperature (*T_c_*, °C), during the cooling cycle at a speed of 10 °C min^−1^. PLA did not completely crystallize during the heating process, as indicated by the occurrence of cold crystallization [8].

In the cooling cycle, the temperature drops below the glass transition temperature, which results in the crystallization of only a part of the sufficiently flexible polymer chains. At temperatures below *T_g_* (°C)*,* macromolecules are in a glassy state and are unable to crystallize completely. Polymer chains that did not have enough time to crystallize during rapid cooling crystallize later during heating at temperatures above the glass transition temperature. After cold crystallization, a melting process occurs, which manifests itself as an endothermic transition with a maximum peak at the melting temperature (*T_m_*, °C) of 152.1 °C. The heating curves of the TPS_SL/PLA blends show a double melting peak, a phenomenon attributed to polylactide since TPS is a completely amorphous polymer. This double melting peak refers to the melting of small and imperfect crystals during the polylactide recrystallization process [37,38]. The first of two approaches for explaining the multiple melting peaks of polylactide upon heating suggests that the double melting peak is the result of the formation of different crystal structures. The structures include a higher-melting α-form and a lower-melting β-form. The β-form is characterized by an imperfect crystal structure and is rarer than the α-form, which dominates as the most widespread polymorph. It is noticeable that the helical conformations of the chains in both forms are influenced by temperature, with the β-form being more sensitive to temperature changes. The α- and β-forms have identical energy levels, which results in their characteristic crystal structure being different in stacking arrangement. The melting-recrystallization concept is another approach used to explain the double melting peak of polylactide, which implies that with an increase in temperature, small and imperfect crystals undergo a melting and recrystallization mechanism to achieve stability [39]. On the curves of polymer blends TPS_SL/PLA in the ratios 60/40 and 50/50, the absence of a double peak can be observed, while on the curve TPS_SL/PLA 40/60 a double peak can be noticed; also visible in polymer blends with the TPS_SL-citrates (Figure 5).

The β-form is formed in a special state of chain motion and stress field that is eliminated by the presence of thermoplastic starch [40]. The α-form is more stable in the presence of a certain proportion of TPS_SL, while the β-form disappears due to limited chain movement [41]. According to the literature, the decrease in the intensity of the melting peak in polymer mixtures is attributed to the morphological effect, while from the thermodynamic point of view, the decrease in the melting temperature is related to the decrease in the chemical potential of the crystalline polymer due to the presence of the amorphous polymer [42]. The lowering of the melting temperature of the blends analysed in this paper can be partially attributed to the mixing with an amorphous polymer, i.e., thermoplastic starch. The arrangement of the crystalline phase is visible from the melting temperature and mostly the degree of order in the crystalline phase corresponds to a higher melting temperature [36]. When the TPS_SL/PLA 60/40 blends with and without citric acid are compared, it is evident that the samples with citric acid have a higher melting temperature compared to the samples without citric acid. Thus, the sample TPS_SL_5CA/PLA 60/40 has the highest Tm (154.2 °C), while the polymer blend without the addition of citric acid (TPS_SL/PLA 60/40) has the lowest Tm (148.4 °C), indicating that the addition of the compatibilizer, in particular citric acid, increases the degree of order in the crystalline phase, i.e., within the polylactide crystal structure. The blending of thermoplastic starch with PLA causes a decrease in melting enthalpy, while the simultaneous decrease in melting enthalpy is accompanied by a shift in cold crystallization temperatures towards lower values, implying that TPS has a nucleating effect on PLA, marked by an acceleration of the cold crystallization process [8]. Polymer blends with TPS_SL-citrates show a reduction in cold crystallization temperature compared to the blends without citric acid, with the samples containing 10 wt.% citric acid showing the most significant reduction in *T_c_* (°C). The polymer blend TPS_SL_10CA/PLA 60/40 has the lowest cold crystallization temperature (104.4 °C), which suggests that the addition of citric acid can accelerate the cold crystallization process. According to the literature, thermoplastic starch simplifies the cold crystallization of PLA, reflecting a more regular arrangement of polylactide chains in the crystal structure [43]. It can be stated that TPS_SL improves the crystallization ability of PLA and enables a more regular arrangement of the polylactide chains in the crystal structure. Although the calculated degree of crystallinity shown in Table 1 shows an improvement in the crystallization of PLA with citric acid (TPS_SL_5CA/PLA 60/40 has the highest value of *χ_c_* of 36.3%), an increase in the citric acid content leads to a decrease in the value of the degree of crystallinity. From the results, it was concluded that the addition of 5 wt.% citric acid has the most favourable effect on the degree of crystallinity of PLA.

#### 3.1.4. Water Vapour Permeability (*WVP*) Test Results

**Figure 6:**
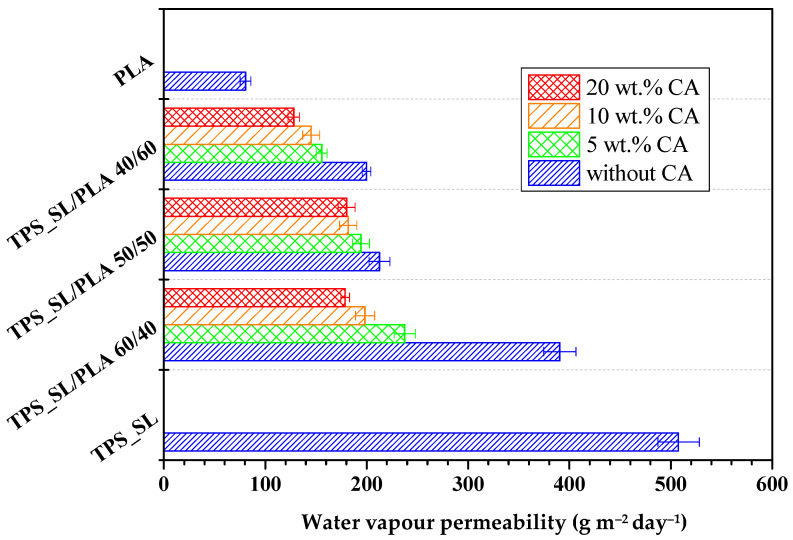
Water vapour permeability of biopolymeric blends.

The obtained results of water vapour permeability (*WVP*) of biodegradable polymer blends TPS_SL/PLA with and without the addition of citric acid are summarized in Figure 6. According to the obtained results, it is evident that pure polylactide shows a low value of water vapour permeability (*WVP* is 80.44 gm^−2^ day^−1^) compared to pure thermoplastic starch, which shows a significantly higher value of water vapour permeability (507.53 gm^−2^ day^−1^). The water vapour permeability values of all tested biodegradable polymer blends are between the water vapour permeability values of pure TPS_SL and PLA. The sample TPS_SL/PLA 40/60 has the lowest *WVP* value, which is attributed to the increase in the PLA content in the blend, while the highest *WVP* value is found in the blend TPS_SL/PLA 60/40. In the sample with TPS_SL-citrates, an increase in the citric acid content leads to a decrease in *WVP*, which leads to the conclusion that the content of 20 wt.% citric acid has the most favorable effect on the barrier properties of the investigated biodegradable polymer blends. As depicted in Figure 6, the results highlight a distinct correlation between the inclusion and increasing concentration of citric acid and a decrease in water vapour permeability (*WVP*) values. This finding emphasizes the role of citric acid in improving the barrier properties of the materials under scrutiny. Particularly noteworthy is the significant reduction in *WVP* values observed at the highest citric acid concentration (20 wt.%) across all investigated TPS_SL/PLA blends.

Higher values of water vapour permeability indicate poor barrier properties related to its ability to pass water vapour and other gases and are important in applications such as the packaging of easily perishable foods where it is desirable for the packaging material to have low values of water vapour permeability to prevent the passage of moisture that could damage the structure of the product. The above-mentioned properties contribute to preserving the durability and safety of the product during its storage and transportation. Due to the reasons mentioned, and in order to improve the barrier properties of thermoplastic starch, mixing with polylactide is carried out and a decrease in water vapour permeability is expected with an increase in the proportion of PLA in TPS_SL/PLA blends. The increase in the proportion of thermoplastic starch leads to an increase in the proportion of the hydrophilic component, which has a greater tendency to absorb water vapour, while on the other hand, PLA contributes to a decrease in permeability due to its pronounced hydrophobic character. With a smaller proportion of TPS, the proportion of voids formed in the structure of TPS_SL/PLA blends as potential water vapour retention spaces are smaller. Glycerol contains free -OH groups, has a hydrophilic nature, and by means of hydrogen bonds binds water molecules, which contributes to an increase in active spaces for the absorption of water molecules, which also contributes to an increase in water vapour permeability [44].

#### 3.1.5. Results of Mechanical Properties

**Figure 7:**
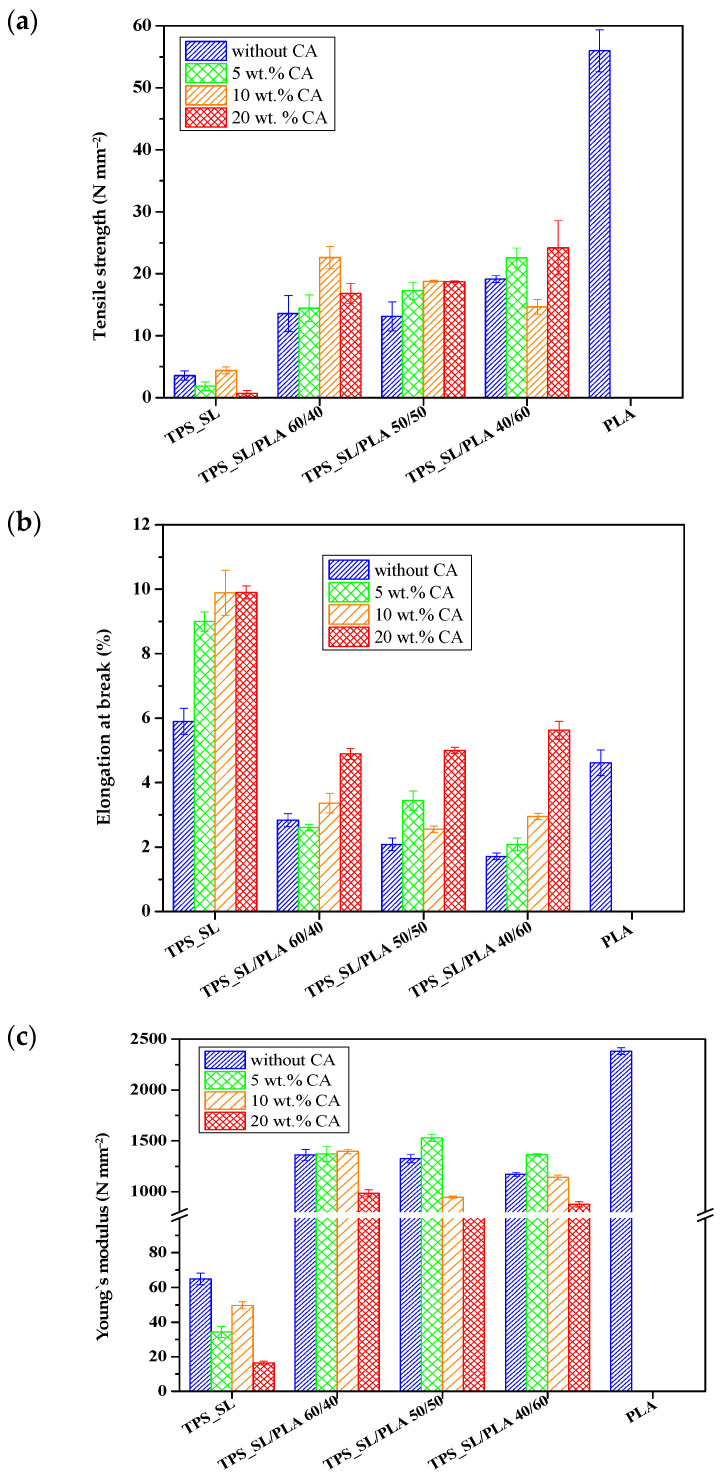
Mechanical properties of biopolymeric blends: (**a**) tensile strength; (**b**) elongation at break; (**c**) Young’s modulus.

Since polymer materials have a wide range of applications, especially in packaging, the requirements for strain values on deformation are increasing; these materials have to be more susceptible to deformation, and thus maintain structural stability [45]. The mechanical properties of TPS_SL, PLA and TPS_SL/PLA polymer blends without and with citric acid are shown in Figure 7: tensile strength (*σ*, Nmm^−2^), elongation at break (*ε*, %) and Young’s modulus (*E*, Nmm^−2^).

Pure polylactide shows high tensile strength values due to its stiffness and brittleness, while pure thermoplastic starch has lower tensile strength and Young’s modulus values and slightly higher elongation at break values compared to polylactide.

The aim of adding polylactide to TPS_SL is to improve the poor mechanical properties of TPS_SL and reduce the stiffness of polylactide. The results of the mechanical properties of biodegradable polymer blends, obtained in this research, are between the values of pure polymers. The application of citric acid in the preparation of thermoplastic starch significantly affects the mechanical properties of TPS_SL, especially the elongation at break. Treatment of TPS_SL with citric acid improves the flexibility of polymer blends due to the ability of citric acid to partially hydrolyse the backbone of TPS_SL, which allows greater stretching of the molecules than with pure TPS_SL. The addition of polylactide to TPS_SL improves the poor mechanical properties of thermoplastic starch while maintaining optimal properties of TPS_SL/PLA blends for specific applications and biodegradability.

### 3.2. Assessing the Biodegradability Potential of Materials

**Figure 8:**
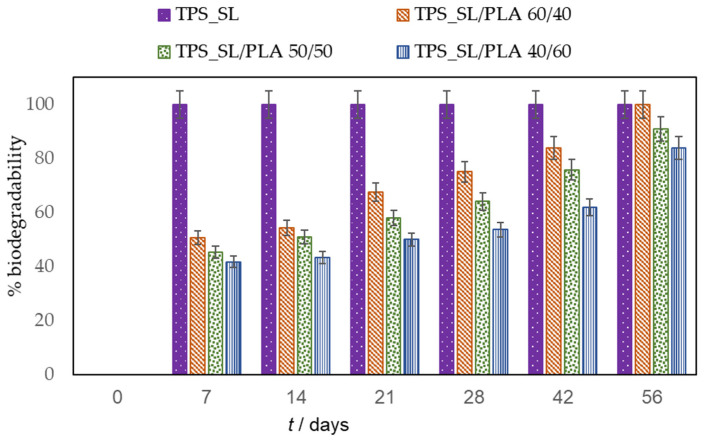
Biodegradability of TPS_SL and TPS_SL/PLA blends.

**Figure 9:**
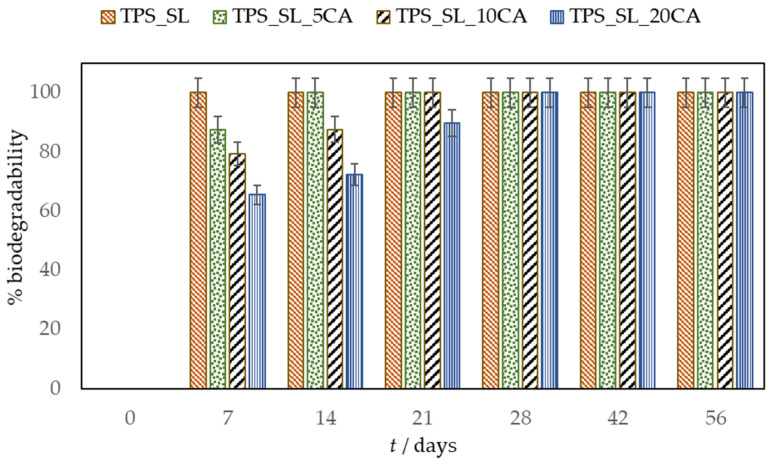
Influence of citric acid (CA) on the biodegradability of TPS_SL.

**Figure 10:**
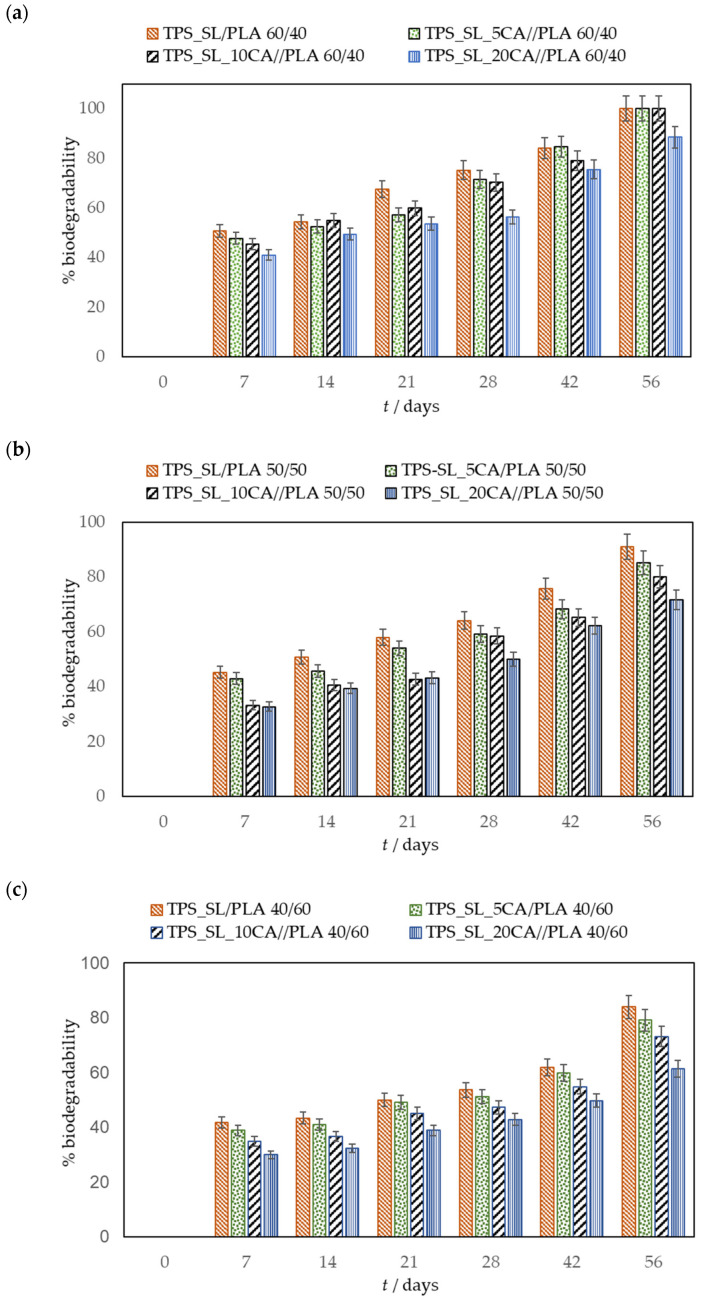
Biodegrability of biopolymeric blends: (**a**) TPS_SL/PLA and TPS_SL-citrate/PLA 60/40; (**b**) TPS_SL/PLA and TPS_SL-citrate/PLA 50/50; (**c**) TPS_SL/PLA and TPS_SL-citrate/PLA 40/60.

This study aimed to determine the biodegradability of blends, comprising thermoplastic starch (TPS_SL) and polylactide (PLA), with a particular focus on evaluating the influence of citric acid on biodegradability (Figure 8, Figure 9 and Figure 10).

**Figure 11:**
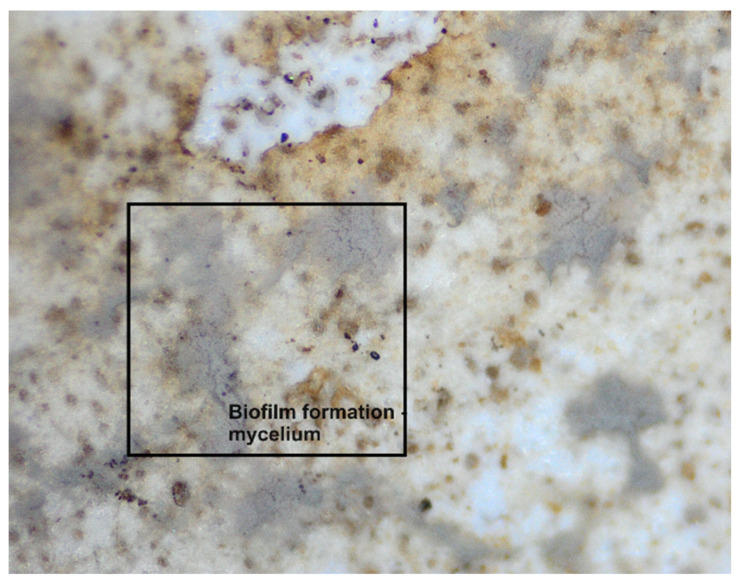
Formation of biofilm of fungi on a TPS_SL/PLA 50/50 polymeric blend in the first 7 days.

During the 56-day process, all prepared samples underwent biodegradation. Pure TPS_SL exhibited complete degradation within 7 days, whereas PLA showed no change in mass over the period of 56 days (Figure 8). Additionally, a notable trend emerged where higher proportions of TPS_SL in the TPS_SL and PLA blends corresponded to more efficient material degradation. For instance, the blend consisting of TPS_SL and PLA in a ratio of 60/40 underwent complete degradation within the 56-day timeframe (Figure 8). Despite the notable increase in degradation rate, significant changes were observed in the appearance of the samples. Particularly, all starch films underwent fragmentation into smaller pieces as a result of the degradation process. Additionally, the polymer experienced fragmentation into lower molecular masses, likely attributed to both abiotic reactions such as oxidation and hydrolysis, as well as biotic reactions involving degradation by microorganisms present in the samples [46]. Subsequently, this fragmentation phase was succeeded by the bioassimilation of the starch fragments by microorganisms and their mineralization. However, prior to the fragmentation of the film, microorganisms formed a biofilm on the polymer surface, signifying the initial phase of biodegradation (Figure 11). This primary phase involves the colonization of the plastic surface by microorganisms, a process strongly influenced by the hydrophobic properties of both the plastic polymer and the microbial surface [47,48,49]. After colonization, microorganisms secrete extracellular enzymes that adhere to the plastic surface. Given that polymer molecules typically lack hydrolysable bonds, these enzymes primarily consist of oxidoreductases capable of oxidizing polymer bonds in the presence of oxygen, metal ions, and ultraviolet radiation.

Samples containing TPS_SL alone, as well as TPS_SL-citrate blends with 5 and 10 wt.% citric acid contents, exhibited complete degradation between the time period of 7 to 21 day, respectively (Figure 9). Conversely, TPS-citrate samples containing 20 wt.% citric acid achieved complete degradation between 28 and 42 days. The incorporation of citric acid into TPS_SL leads to a decelerated degradation of TPS_SL. This trend is also noticeable in TPS_SL/PLA polymer blends when citric acid is added (Figure 10a–c). The biodegradability of the material decreases with the addition of citric acid, particularly when the concentration exceeds 10 wt.%. This can be attributed to the mechanism where moisture absorption from the soil, coupled with the presence of microorganisms, causes a reduction in the mechanical strength of TPS_SL. Specifically, water induces swelling of TPS_SL, thereby facilitating biodegradation. However, the presence of citric acid diminishes the swelling of the sample, consequently hindering microbial attack due to enhanced moisture resistance. As a result, the analysis of percentage weight loss indicates an improved degradation rate of TPS_SL compared to TPS_SL-citrate, likely attributable to the robust hydrogen interaction between the hydroxyl group of starch molecules and the citric acid, facilitated by the chemical mechanical hydrolysis process [50]. However, the addition of citric acid can also have positive effects on the biodegradability of polymer mixtures. For instance, Ibrahim et al. observed the impact of citric acid on the TPS_SL/PLA mixture and found that introducing 6 wt.% citric acid enhances the biodegradation process of these polymer blends [25]. This observed enhancement can be attributed to the substantial presence of citric acid in the polymer blend, which leads to the hydrolysis of TPS chains and consequently promotes TPS degradation through an acidosis mechanism. However, in the present study, it is evident that the incorporation of 5 wt.% citric acid had no significant impact on the material’s biodegradability. The results obtained suggest that the impact of citric acid on biodegradation is contingent upon its proportion in the mixture of polymeric materials. It has been observed that citric acid can either accelerate or decelerate the biodegradation process.

Figure 10 illustrates the impact of the TPS_SL proportions on the biodegradability of TPS_SL/PLA films. Analysis of the data reveals that the TPS_SL proportion in the polymer blends plays a significant role in biodegradation. Specifically, polymer blends with a higher TPS_SL proportion exhibit a greater change in mass compared to those with a lower TPS_SL proportion. This phenomenon can be attributed to the fact that biodegradation typically occurs more rapidly in the amorphous regions of the polymer, as microorganisms find it easier to attack these areas. In contrast to TPS_SL, an increase in the proportion of PLA results in a decrease in biodegradation.

**Figure 12:**
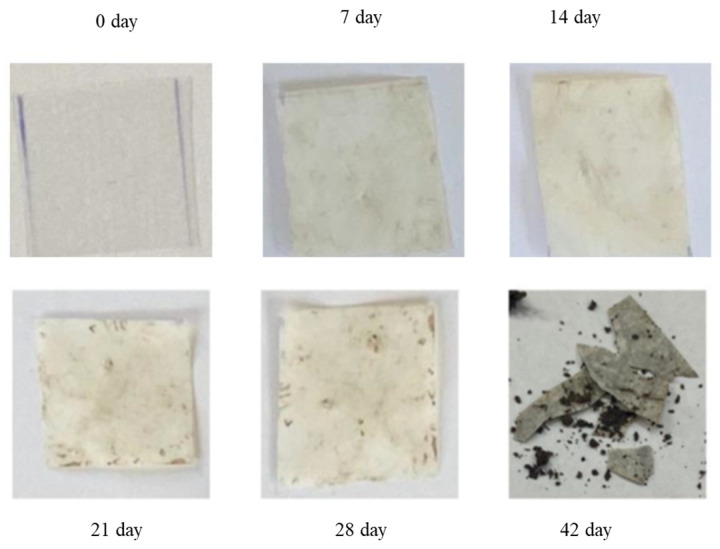
Biodegradation of pure PLA.

This can be explained by the presence of semi-crystalline PLA in the blends, which reduces the amorphous regions. Consequently, the molecular weight increases significantly, and the crystalline regions start to decrease, leading to material fragmentation [51,52]. Upon comparing the samples depicted in Figure 1, it was noted that the pure PLA sample did not exhibit any structural breakage or formation of a biofilm. The surface of the pure PLA appears uniform, showing no apparent signs of biodegradation which is consistent with published research [53]. However, upon removing the sample from the soil on the 42nd day, it broke (Figure 12). This is likely attributed to the material’s brittleness and its propensity to absorb moisture.

**Figure 13:**
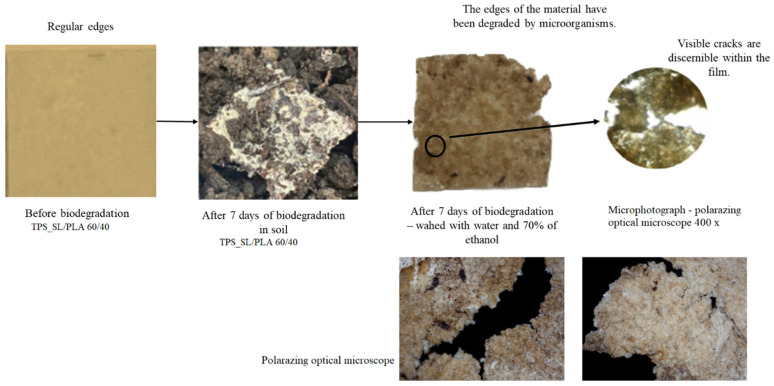
Morphological observation of the prepared TPS_SL/PLA 60/40 film by polarizing optical microscope before and after 7 days of biodegradation.

**Figure 14:**
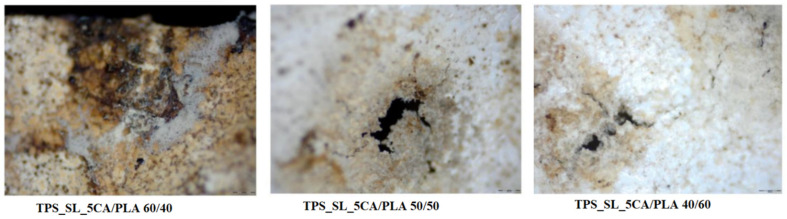
Surface microstructure of the TPS_SL_5CA/PLA blends after 7 days of biodegradation using a polarizing optical microscope (100×).

**Figure 15:**
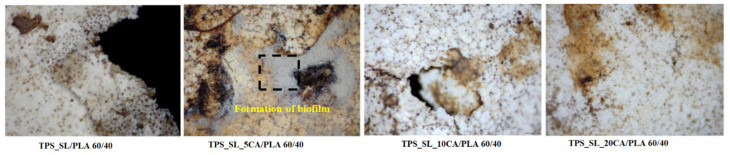
Surface microstructure of the TPS_SL_(5–20)_CA/PLA 60/40 samples after 7 days of biodegradation using a polarizing optical microscope (100×).

The changes observed in the surface morphology of the samples by optical and polarizing microscope are illustrated in Figure 13, Figure 14 and Figure 15. Optical photographs depicting TPS_SL and PLA, along with the addition of citric acid after 7 days of soil burial tests, reveal noticeable alterations in their tonality, as well as the emergence of voids and cracks. Figure 6 depicts the TPS_SL/PLA film 60/40 both before and after biodegradation, alongside a micrograph capturing the sample, exhibiting distinct porous regions. The rapid degradation of TPS_SL results in the formation of porous areas, or voids, within the structure. These voids play a pivotal role by enhancing access for microorganisms, thereby catalyzing the biodegradation process of other components within the polymer blend [54]. The depicted images also illustrate the stages of polymer degradation, namely biodeterioration, biofragmentation, and assimilation. Among these stages, biodeterioration emerges as the most prominent, marked by the proliferation of microorganisms on the polymer’s surface, leading to notable changes in its mechanical, chemical, and physical characteristics. During biodeterioration, bacteria secrete a viscous substance that permeates the polymer’s surface, while fungi develop a network of mycelia (as shown in Figure 14 and Figure 15). These microorganisms are capable of infiltrating pores within the polymer structure, causing them to expand and facilitating the formation of cracks. This process illustrates the intricate interactions between microorganisms and polymers, highlighting the dynamic nature of biodeterioration and its significant impact on the degradation process.

Polymer blends with a lower content of polylactic acid (PLA) exhibit greater susceptibility to biodegradation compared to those with a higher PLA content, as can be seen in Figure 14. This observation can be attributed to the slower biodegradation rate of blends with higher PLA content, which is primarily influenced by the slow biodegradation of pure polylactide due to its high crystallinity and pronounced hydrophobic nature [55]. Furthermore, the presence of PLA in higher proportions hinders deeper penetration of microorganisms, thus making it more challenging for them to access the TPS_SL component, which is hydrophilic. Consequently, blends with a higher TPS_SL content manifest more visible areas of initial biodegradation on the surface, characterized by irregular edges and cracks, compared to blends with lower TPS_SL content, where structural damage is less severe. When the microphotographs in Figure 15 are compared, it is evident that the TPS_SL/PLA 60/40 sample undergoes more extensive degradation compared to samples containing citrate. After just 7 days of biodegradation, this sample exhibits greater structural damage, characterized by an increased number of cracks, voids, and noticeable colour changes. This discrepancy can be attributed to the ease with which hydrogen bonds between starch and glycerol (present in TPS_SL-citrate) break in comparison to those between starch and carboxylic acid. Zain et al. (2017) demonstrated that the addition of citric acid (forming TPS citrate) enhances resistance to moisture absorption, suggesting that the formation of TPS citrate significantly influences the biodegradation process [50]. Similar findings were reported by Seligr et al. (2016), who observed that citric acid inhibits microbial attack due to its superior resistance to moisture uptake compared to samples lacking citric acid [56].

#### Exploring Material Transformation Post-Biodegradation through FTIR Analysis

**Figure 16:**
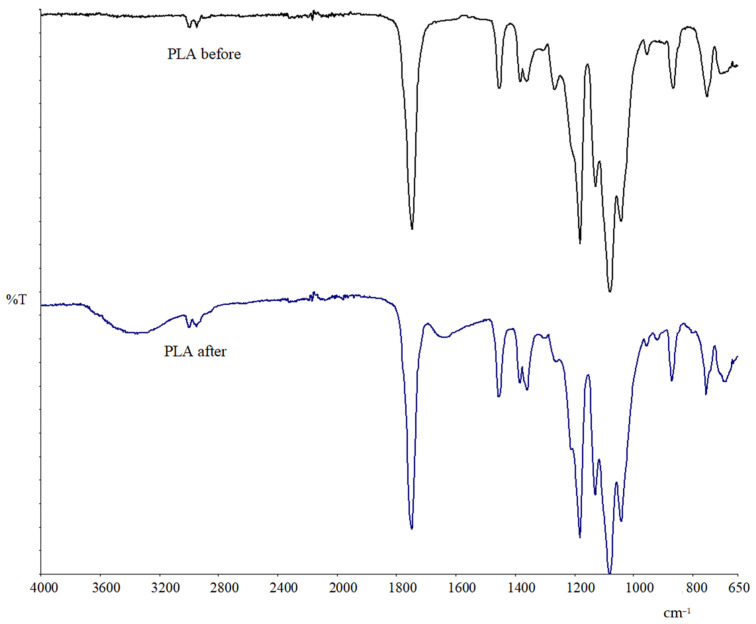
FTIR spectra of PLA before and after biodegradation.

The structural changes occurring in both TPS and TPS/PLA before and after soil burial were subjected to further analysis using FTIR. The aim of the analysis was to investigate how the biodegradation process affects pure PLA and TPS_SL/PLA polymer blends. The characteristic vibrational bands associated with ester bonds are prominently displayed in the spectrum of polylactide, which falls within the polyester group. In Figure 16, depicting the spectrum of pure polylactide before and after biodegradation, the initial signals are observed at 2997 cm^−1^ and 2941 cm^−1^, representing the symmetric stretching of the C-H bond in the methyl group.

Additionally, distinct peaks indicating the stretching of the C=O bond are evident at 1748 cm^−1^, along with two peaks indicating the bending of the O-H bond between 1383 cm^−1^ and 1360 cm^−1^. Following biodegradation, most of the characteristic peaks of PLA remain unchanged. However, a novel peak emerges at 3347 cm^−1^, which is a characteristic of the stretching of the O-H group, likely attributed to bound water—indicating moisture absorption by the sample during biodegradation in soil. Nevertheless, a notable shift of peaks towards higher wavenumbers accompanied by a reduction in intensity is observed in the FTIR spectrum. Specifically, the shift of the peak corresponding to the carbonyl group from 1746 to 1751 cm^−1^ suggests potential degradation of PLA, primarily due to ester bond hydrolysis in the presence of microorganisms. This observation aligns with findings by Agarwal et al. (1998) [57], confirming the propensity of PLA degradation via ester bond hydrolysis mediated by microbial activity.

**Figure 17:**
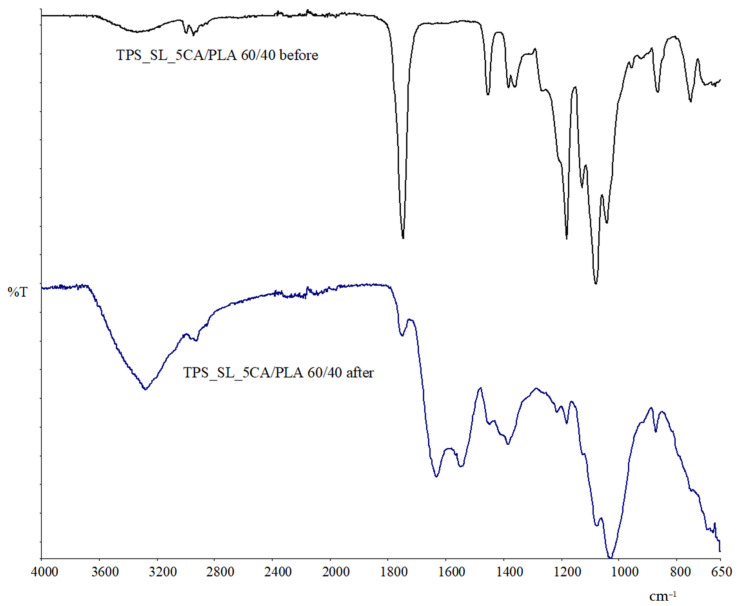
FTIR spectra of TPS_SL_5CA/PLA 60/40 before and after biodegradation.

The FTIR spectrum of the TPS_SL_5CA/PLA 60/40 film after biodegradation (Figure 17) reveals a significant absence of peaks within the range of 3330 cm^−1^ to 3280 cm^−1^, which corresponds to the hydroxyl (OH) group of starch. This absence indicates the degradation of starch within the TPS_SL/PLA blend [58]. However, the presence of the C=O peak, visible at 1747 cm^−1^, is consistent across all polymer blends, confirming that degradation occurred solely within the starch structure and not within the PLA component within the span of 56 days of biodegradation [58]. This observation underscores the selective degradation of starch, leaving the PLA structure unaffected during the degradation process.

In the study by Palai et al. (2021), FTIR spectra of polymer blends of PLA and polybutylene succinate adipate (PBSA) exhibited characteristic peaks associated with the stretching and bending of different chemical groups [58]. Although the FTIR spectra of samples buried in soil showed peak shifts, these shifts did not significantly change before and after 90 days in the soil. However, the observed shifts in peaks and reduction in intensity indicated degradation of the PLA/PBSA film in the soil, a trend consistent with findings obtained by Weng et al. (2013) on PLA/PBAT blends [59]. Before biodegradation, the FTIR spectra of the PLA/TPS system displayed peaks related to the stretching and bending of starch and PLA [59]. Subsequent to degradation, the absence of peaks in the hydroxyl group (OH) region indicated degradation of the starch component of the PLA/TPS polymer blend, while the peak corresponding to carbonyl stretch remained relatively unchanged, signifying the stability of PLA within 90 days of soil exposure. These findings support observations by Akrami et al. (2016), who noted the absence of a hydroxyl peak and an unchanged carbonyl peak during the biodegradation of PLA and corn starch samples in soil [41]. Structural changes were detected when the film underwent biodegradation, with observable alterations in the spectra attributed to changes in starch crystallinity reflected in the glycosidic bonds of TPS. Starch degradation, occurring primarily in the amorphous region (amylose), as well as in PLA where hydrogen bonds are weak and more accessible to microorganisms, suggests faster degradation in the amorphous compared to the crystalline part. This emphasizes the pivotal role of accessibility to microorganisms in the degradation process.

## 4. Conclusions

FTIR-ATR spectroscopy proved the formation of an ester bond visible on the basis of the appearance of new absorption bands, which is a characteristic of such compounds. Ester bonds were proven in all ratios of the investigated biodegradable TPS_SL/PLA polymer blends due to the reaction of citric acid with the –OH group from starch. SEM micrographs of TPS_SL/PLA polymer blends indicate weak adhesion between the polylactide matrix and thermoplastic starch. With the addition of 5 wt.% citric acid to TPS_SL, an improvement in the miscibility of the analysed biodegradable TPS_SL/PLA blends was observed, which also improves the homogeneity of the mixtures. DSC analysis confirmed that thermoplastic starch can accelerate the process of cold crystallization and nucleation effect on polylactide. An increase in the proportion of TPS_SL in the blends results in a higher degree of crystallinity of PLA, while a lower proportion of added citric acid affects the growth of the degree of crystallinity. It has been proven that citric acid in TPS_SL/PLA blends reduces the glass transition temperature, further accelerates the cold crystallization process of PLA, and confirms its purpose as a compatibilizer of polymer blends. Water vapour permeability increases as expected with an increase in TPS_SL content of the TPS_SL/PLA blends. With an increase in the proportion of citric acid, a decrease in *WVP* is observed, which leads to the conclusion that the proportion of 20 wt.% most favorably affects the barrier properties of the analysed biodegradable polymer blends. Citric acid has a positive effect on the mechanical properties of polymer blends by increasing the elongation at break of TPS, while simultaneously reducing the stiffness of biodegradable polymer blends with the addition of polylactide. Good mechanical properties are associated with SEM micrographs where good adhesion between thermoplastic starch and polylactide can be observed as well as homogeneous distribution of TPS_SL within the polylactide matrix with the addition of citric acid at a proportion of 5 wt.%. Through the process of biodegradation, it became evident that higher proportions of TPS in polymer blends notably hasten the degradation of TPS/PLA compositions. However, upon the addition of citric acid, there was a discernible slowdown in the biodegradation rate of these polymer mixtures, attributed to its inhibitory effect on microbial growth.

## Figures and Tables

**Table 1:** Thermal properties of biopolymeric blends.

Sample	*T_g_* (°C)	*T_c_* (°C)	Δ*H_c_* (Jg^−1^)	*T_m_* (°C)	Δ*H_m_* (Jg^−1^)	*χ_c_* (%)
PLA	58.5	112.4	20.9	152.1	20.2	21.7
TPS_SL/PLA 60/40	56.7	114.4	13.0	148.4	12.5	33.6
TPS_SL/PLA 50/50	56.6	116.1	14.4	148.9	14.1	30.2
TPS_SL/PLA 40/60	54.9	110.8	21.3	153.3	20.2	36.2
TPS_SL_5CA/PLA 60/40	56.2	111.3	13.6	154.2	13.5	36.3
TPS_SL_5CA/PLA 50/50	55.3	110.2	16.4	153.1	15.3	32.9
TPS_SL_5CA/PLA 40/60	55.1	110.4	20.0	153.7	19.2	34.4
TPS_SL_10CA/PLA 60/40	55.9	104.4	13.2	153.3	11.7	31.4
TPS_SL_10CA/PLA 50/50	55.2	104.6	15.8	152.7	14.1	30.3
TPS_SL_10CA/PLA 40/60	54.5	109.9	17.6	153.6	18.8	33.6
TPS_SL_20CA/PLA 60/40	54.6	110.5	13.5	153.8	12.4	33.4
TPS_SL_20CA/PLA 50/50	53.8	110.1	17.1	153.3	16.4	35.2
TPS_SL_20CA/PLA 40/60	55.4	109.7	20.1	155.3	19.5	34.8

*T_g_—*glass transition temperature; *T_c_—*crystallization temperature; *T_m_—*melting temperature; Δ*H_c_—*crystallization enthalpy; ∆*H_m_—*melting enthalpy; *χ_c_—*degree of crystallinity.

## Data Availability

Data are contained within the article.
